# Comprehensive Analysis of APA Events and Their Association With Tumor Microenvironment in Lung Adenocarcinoma

**DOI:** 10.3389/fgene.2021.645360

**Published:** 2021-03-18

**Authors:** Yuchu Zhang, Libing Shen, Qili Shi, Guofang Zhao, Fajiu Wang

**Affiliations:** ^1^Department of Intensive Care Medicine, HwaMei Hospital, University of Chinese Academy of Sciences, Ningbo, China; ^2^Ningbo Institute of Life and Health Industry, University of Chinese Academy of Sciences, Ningbo, China; ^3^Institute of Neuroscience, Center for Excellence in Brain Science and Intelligence Technology, Chinese Academy of Sciences, Shanghai, China; ^4^Fudan University Shanghai Cancer Center and Institutes of Biomedical Sciences, Shanghai Medical College, Fudan University, Shanghai, China; ^5^Department of Cardiothoracic Surgery, HwaMei Hospital, University of Chinese Academy of Sciences, Ningbo, China

**Keywords:** alternative polyadenylation, lung adenocarcinoma, immunity, metabolism, miRNA

## Abstract

**Background:**

Alternative polyadenylation (APA) is a pervasive posttranscriptional mechanism regulating gene expression. However, the specific dysregulation of APA events and its potential biological or clinical significance in lung adenocarcinoma (LUAD) remain unclear.

**Methods:**

Here, we collected RNA-Seq data from two independent datasets: GSE40419 (*n* = 146) and The Cancer Genome Atlas (TCGA) LUAD (*n* = 542). The DaPars algorithm was employed to characterize the APA profiles in tumor and normal samples. Spearman correlation was used to assess the effects of APA regulators on 3′ UTR changes in tumors. The Cox proportional hazard model was used to identify clinically relevant APA events and regulators. We stratified 512 patients with LUAD in the TCGA cohort through consensus clustering based on the expression of APA factors.

**Findings:**

We identified remarkably consistent alternative 3′ UTR isoforms between the two cohorts, most of which were shortened in LUAD. Our analyses further suggested that aberrant usage of proximal polyA sites resulted in escape from miRNA binding, thus increasing gene expression. Notably, we found that the 3′ UTR lengths of the mRNA transcriptome were correlated with the expression levels of APA factors. We further identified that CPSF2 and CPEB3 may serve as key regulators in both datasets. Finally, four LUAD subtypes according to different APA factor expression patterns displayed distinct clinical results and oncogenic features related to tumor microenvironment including immune, metabolic, and hypoxic status.

**Interpretation:**

Our analyses characterize the APA profiles among patients with LUAD and identify two key regulators for APA events in LUAD, CPSF2 and CPEB3, which could serve as the potential prognostic genes in LUAD.

## Introduction

Non-small cell lung cancer (NSCLC) is the leading cause of cancer-related mortality worldwide (Herbst et al., 2018). Lung adenocarcinoma (LUAD) is the most prevalent histologic subtype of NSCLC and accounts for approximately 40% of all lung cancer cases (Zappa and Mousa, 2016). The 5-year survival rate for LUAD still remains poor, owing to the dismal prognosis and limited effective treatments. Therefore, elucidating the potential molecular mechanisms underlying LUAD is necessary. Advances in the characterization of alterations in the LUAD transcriptome facilitate interpretations of the complexity of the RNA processing-associated events, such as alternative splicing and polyadenylation, thus providing new perspectives on the oncogenic processes and signaling pathways in cancer development and progression (Esfahani et al., 2019).

Alternative polyadenylation (APA) has been recognized as an important factor regulating gene expression. Approximately, 70% of known human genes contain multiple polyA sites, which produce different lengths of 3′ untranslated regions (3′ UTR), thereby contributing to transcriptome diversity (Derti et al., 2012). 3′ UTR accommodates *cis* elements such as AU-rich elements (Halees et al., 2008) and microRNA (miRNA)-binding sites (Lin et al., 2012), which are involved in various aspects of posttranscriptional RNA processing. Thus, alternative usage of polyA sites can affect mRNA stability, translation, and cellular localization (Tian and Manley, 2017). The polyadenylation of mRNAs is driven by approximately 20 core proteins comprising four complexes: cleavage and polyadenylation specificity factor, cleavage stimulation factor (CstF), cleavage factors I and II, and several single proteins (Gruber and Zavolan, 2019).

Widespread shortening of 3′ UTRs has been identified in multiple types of cancer (Xia et al., 2014; Xiang et al., 2018) and cancer cells (Mayr and Bartel, 2009); this shortening activates oncogenes (Masamha et al., 2014) or represses tumor-suppressor genes (TSGs) in *trans* via disruption of the ceRNA (competing endogenous RNA) network (Park et al., 2018), thus promoting tumorigenesis. Perturbations in the expression level of APA factors have been frequently observed in a variety of cancer types, resulting in aberrant usage of proximal polyA sites (PAS) (Tan et al., 2018; Chu et al., 2019; Fischl et al., 2019; Xiong et al., 2019). Several computational tools utilizing standard RNA-sequencing (RNA-Seq) data for global APA profiling have been developed (Xia et al., 2014; Arefeen et al., 2018; Ye et al., 2018) that facilitate the identification of recurrent and tumor-specific APA events across human cancers (Xia et al., 2014; Xiang et al., 2018; Venkat et al., 2020). Nevertheless, in-depth analysis of specific APA changes in LUAD and their biological or clinical significance in a sufficiently large cohort remain to be determined. To this end, we gathered a large collection of RNA-Seq data from two LUAD cohorts, GSE40419 and TCGA-LUAD, and analyze their differences and similarities in PAS usage. We performed a systematical analysis to reveal the potential regulation and effects of APA in LUAD.

## Materials and Methods

### Data Collection

RNA-Seq data and the corresponding clinical information from two independent LUAD cohorts including tumor and normal samples were downloaded from the TCGA data portal^1^ and NCBI Gene Expression Omnibus (GEO) under accession number GSE40419. The numbers of paired samples in those two sets were 57 and 73 for differential analysis. The TCGA dataset contained 484 tumor samples for subsequent analyses. RNAs used in the TCGA dataset were polyA enriched and those in the GSE40419 dataset were unspecified.

### Characterization of APA Events

The DaPars algorithm^2^ was employed to quantify the relative polyA site usage in 3′ UTR resulting from APA through the Percentage of Distal polyA site Usage Index (PDUI), which indicates lengthening (positive index) or shortening (negative index) of 3′ UTRs (Xia et al., 2014). To identify the differences in 3′ UTRs between tumor and normal samples, we utilized the paired Wilcoxon rank-sum test to determine the significance. The differential APA events were defined by the Benjamini–Hochberg adjusted *p*-value (i.e., false discovery rate) <0.05 and |ΔPDUI| = |PDUI_tumor_ − PDUI_normal_| > 0.1.

### Analysis of miRNA-Binding Sites and DEGs

miRNA-predicted targets and binding sites were downloaded from TargetScanHuman 7.2. High-confidence sites were filtered by context + score percentile > 90 (Agarwal et al., 2015). We then applied this genomic feature on the 3′ UTR changes identified by the DaPars algorithm to acquire the number of genes that lost miRNA targets. The R package “EdgeR” (version 3.30.3) was employed to identify differentially expressed genes (DEGs) with a Benjamini and Hochberg adjusted *p*-value < 0.05 (Robinson et al., 2010).

### Analysis of APA Core Regulators

Genes in the GO terms associated with mRNA polyadenylation (mRNA polyadenylation, mitochondrial mRNA polyadenylation, regulation of mRNA polyadenylation, negative regulation of mRNA polyadenylation, and positive regulation of mRNA polyadenylation) were considered as APA core regulators. All the somatic mutations of the TCGA-LUAD cohort were obtained from the publicly available TCGA MAF file which includes 562 patients [3]. This dataset along with the copy number variation data were directly downloaded from cBioPortal^3^ (Gao et al., 2013). APA regulator genes were expected to control the 3′ UTR lengths of targets. The expression levels of those regulators can be influenced by the copy number variation. Therefore, the transcripts per kilobase million (TPM) values for APA regulators were used to calculate the Spearman correlations between each PDUI and the copy number change in those regulators in tumors. A Spearman correlation coefficient | rho| > 0.3 and adjusted *p*-value < 0.05 were considered significant.

### Survival Analysis for APA Events and Their Regulators

The univariate Cox proportional-hazard model implemented in the “coxph” function from the R package “survival” (version 3.1-12) was used for each differential APA event and regulator gene. The expression levels of APA regulators were log2(TPM + 0.01) transformed before analysis. A likelihood ratio test with *p* < 0.05 was considered significantly associated with survival time. Hazard ratios >1 indicated survival risks, whereas those <1 were associated with better outcomes.

### Clustering Samples Based on Transcriptional Profiles of APA Regulators

*Z*-score transformation was performed to normalize the expression of 35 APA regulators. Consensus *K*-means clustering of 512 LUAD samples on the basis of the Euclidean distances of the APA regulators was conducted from *k* = 2 to *k* = 9. For each iteration, 80% of the tumor samples and 100% of the regulators were selected. This process was repeated for 1,000 times. Empirical cumulative distribution CDF plots were generated for each *k* to identify the *k* at which the CDF area reached an approximate maximum value. This clustering analysis was performed in the R package “ConsensusClusterPlus” (version 1.52.0) (Wilkerson and Hayes, 2010). Kaplan–Meier survival curve analysis and log-rank tests were used to compare the survival distributions among the four groups identified by consensus clustering in the R package “survival” (version 3.1-12).

### Calculation of Immune, Hypoxic, and Metabolic Enrichment Scores

Gene markers of 22 immune cells were downloaded from CIBERSORT^4^ (Newman et al., 2015). A 15-gene expression signature was selected for the hypoxia markers because they have been shown to classify hypoxia status at best (Ye et al., 2019). Single sample gene set enrichment analysis (ssGSEA) implemented in the R package “GSVA” (version 1.24.0) (Hanzelmann et al., 2013) was conducted to calculate the normalized enrichment score (NES) for each gene set of the 22 immune cells and the hypoxia status. Genes of 5 metabolic pathways were downloaded from the Kyoto Encyclopedia of Genes and Genomes (KEGG) database. Gene set variation analysis (GSVA) was used to calculate the enrichment score of each metabolic pathway.

## Results

### Global 3′ UTR Shortening in LUAD

**FIGURE 1:**
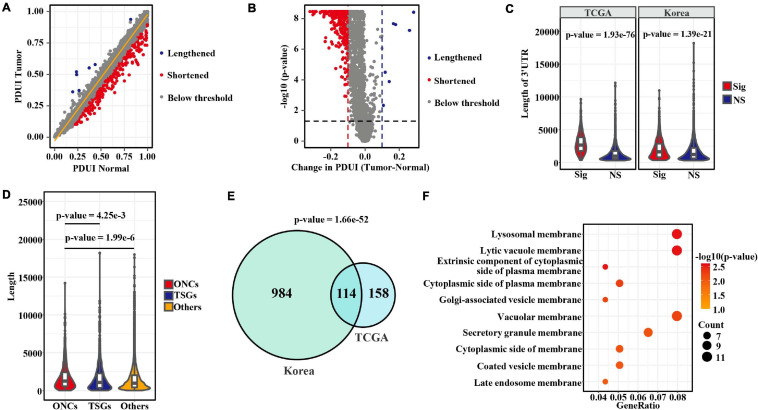
Comprehensive characterization of aberrant APA in LUAD. **(A)** Scatterplot of PDUIs in normal (*x* axis) and tumor (*y* axis) samples from the TCGA cohort. Significantly (adjusted *p*-value < 0.05 and | ΔPDUI| > 0.1) shortened and lengthened transcripts are indicated in red and blue, respectively, whereas those below the threshold are gray. **(B)** Volcano plot showing the significantly altered APA events in the TCGA cohort. **(C)** Comparison of 3′ UTR lengths between significantly changed transcripts in tumors and other transcripts detected in both tumor and normal samples that did not pass the threshold. Here, *p*-values were calculated by the Wilcoxon rank-sum test. **(D)** Comparison of 3′ UTR lengths among oncogenes, tumor-suppressor genes, and other genes annotated in databases. Statistical differences were determined by the Wilcoxon rank-sum test. **(E)** Venn diagram showing the strong overlap of altered APA events between the two datasets. **(F)** Dot plot indicating significantly enriched cellular components of genes with recurrent APA alterations found in both two cohorts.

To explore the APA changes between tumor and adjacent normal samples, we analyzed 57 and 73 paired patients with LUAD from the TCGA and Korean cohorts, respectively. Among the events detected in the tumor group or the normal group, less than half of samples (occurrence rate < 50%) were discarded. A total of 4,303 and 7,267 events remained for differential analysis in those two sets. Among those events, 272 and 1,098 from 263 to 1,074 genes significantly differed (adjusted *p*-value < 0.05 and | PDUItumor − PDUInormal| > 0.1) in the TCGA and Korean datasets, respectively (Figures 1A,B and Supplementary Figures 1A,B). Notably, the numbers of shortened 3′ UTR events in tumors far exceeded the numbers of lengthened 3′ UTR events (Figures 1A,B and Supplementary Figures 1A,B), in agreement with the patterns observed in previous pan-cancer analyses (Xia et al., 2014; Xiang et al., 2018). The significantly changed transcripts showed longer 3′ UTR lengths than were observed below the threshold (Figure 1C). Furthermore, we compared the 3′ UTR lengths among oncogenes (Liu et al., 2017), TSGs (Zhao et al., 2016), and other genes (Figure 1D). The results indicated that oncogenes tended to have longer 3′ UTR length than TSGs and other genes. Next, to determine the recurrent APA alterations in LUAD, we combined the results from the two studies. As shown in Figure 1E. A total 114 transcripts were determined to have changed in both cohorts, thus representing a strongly significant overlap (*p*-value = 1.66e−52, hypergeometric test). APA-derived 3′ UTRs have been proposed to affect the mRNA and protein location (Berkovits and Mayr, 2015). Therefore, we conducted an overrepresentation analysis of cellular component for those recurrent APA alterations found in LUAD by using the R package “clusterProfiler” (version 3.11) (Yu et al., 2012). Strikingly, the recurrent changed genes were highly enriched in the membrane (Figure 1F), thus suggesting that APA may be involved in regulating the localization of membrane proteins (Berkovits and Mayr, 2015) or the subcellular localization of mRNA transcripts for cancer-specific genes. For example, we showed the detailed recurrent alterations in 3′ UTRs located in lysosomal membranes (Supplementary Figures 1C–F).

### 3′ UTR Shortening-Mediated Loss of miRNA-Binding Sites

**FIGURE 2:**
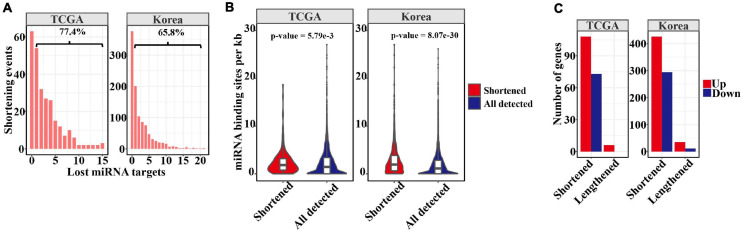
APA mediated loss of miRNA binding sites. **(A)** Barplots showing the distribution of lost miRNA-binding sites resulting from 3′ UTR shortening. The percentage of shortening events losing at least one miRNA-binding site is displayed above the bracket. **(B)** Comparison of miRNA-binding sites between significantly shortened transcripts in tumors and others below the threshold. **(C)** Barplots showing the number of upregulated or downregulated that may be affected by APA in tumors.

Independently of mRNA and protein localization, 3′ UTR shortening through APA during tumorigenesis may escape miRNA repression, thus increasing gene expression. Therefore, we calculated the distribution of lost miRNA-binding sites as a result of shortened 3′ UTR lengths in LUAD (Figure 2A). As revealed by this analysis, 77.4 and 65.8% of events with shortened 3′ UTRs from the TCGA and Korean cohorts had lost at least one predicted miRNA-binding site (Figure 2A). Furthermore, we compared the miRNA-binding sites for 3′ UTR shortened transcripts with those below the threshold. Consistent with a previous pan-cancer analysis (Xia et al., 2014), the results (Figure 2B) showed that those shortened events in tumors had overall greater miRNA-binding site density (*p*-value = 1.34e−10 and 0, Kolmogorov–Smirnov test), suggesting that cancer cells may maximize the mitigation of miRNA binding by preferentially shortening the 3′ UTR, in a process strictly regulated by miRNAs. To examine the effects of miRNA-binding loss mediated by 3′ UTR shortening, we analyzed DEGs between paired normal and tumor tissues. Among genes with shortened 3′ UTR, 103 and 417 were significantly upregulated in the tumors in the two cohorts, possibly as a consequence of escape from miRNA repression (Figure 2C). Nevertheless, when compared with all DEGs, the genes with shortened 3′ UTRs did not tend to be more upregulated in LUAD (*p*-value = 0.07 and 1, hypergeometric test). This result is consistent with prior analyses in pancreatic ductal adenocarcinoma (Venkat et al., 2020) and other types of cancer (Xiang et al., 2018), suggesting the presence of other mechanisms in regulating gene expression. In addition, we found three genes, COL5A1, COL1A2, and CP with lengthened 3′ UTR were upregulated in tumors in both the datasets. Several genes have been reported that their longer 3′ UTR isoform can enhance expression through *trans*-regulation mechanism (Allen et al., 2013; Arake et al., 2019).

### Regulators of APA Events in LUAD

**FIGURE 3:**
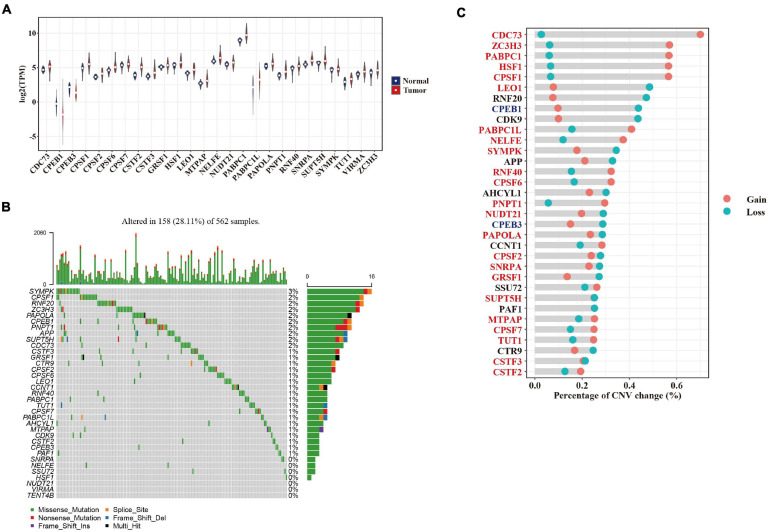
Genetic and expression alterations in APA regulators. **(A)** Violin plot showing the expression of 26 significantly dysregulated APA factors between tumor (red) and adjacent normal (blue) samples in the TCGA cohort. **(B)** The mutation landscape of 35 APA regulators in the TCGA cohort. Top panel shows the tumor mutation rate of each patient. Bottom panel indicates the mutation frequency of individual regulators. Mutation types are shown in the legend at the bottom. **(C)** The CNV variation frequency of APA regulators in the TCGA cohort. Gain and loss of CNV are indicated by red and blue dots, respectively. Upregulated and downregulated APA factors are colored in red and blue.

To investigate potential regulators governing APA alternations in LUAD development, we analyzed the differential expression of 35 genes collected from the GO terms associated with “mRNA polyadenylation” between normal and tumor sample pairs. Most of those genes (TCGA, 26/35, and Korean, 25/32) that were differentially expressed (adjusted *p*-value < 0.05) in the two cohorts were upregulated in tumors (Figure 3A and Supplementary Figure 2A). Moreover, we found that 19 and 2 APA regulators were both upregulated and downregulated in two datasets (Figure 3A and Supplementary Figure 2A). For example, CSTF2 has been reported to promote 3′ UTR shortening of cancer-related genes in NSCLC and was upregulated in both the TCGA and Korean cohorts. To further explore genetic alterations in APA regulators in LUAD that may affect their expression levels, we analyzed somatic mutations and copy number variations (CNVs) of these genes in the TCGA cohort. We found that 28.1% (158/562) of the LUAD tumor samples had at least one protein-affecting mutation (Figure 3B). Most components of the 3′ end-processing machinery are RNA-binding proteins; the mutation frequency of these factors ranged from 0.2 to 2.8%, a percentage not greater than that for other RNA-binding proteins observed in pan-cancer studies (Sebestyen et al., 2016; Li et al., 2019). Compared with somatic mutations, CNVs were highly recurrent across patients with a range of 32.1–72.8% (Figure 3C). We found that 69.7% (23/33) of APA factors were positively correlated (rho > 0.3 and adjusted *p*-value < 0.05) with their mRNA expressions in tumors (Supplementary Figure 2B). A total of 14 APA regulators with more than half of CNV gains showed significantly higher expression in tumors (e.g., CDC73 and ZC3H3), whereas the two downregulated factors, CPEB1 and CPEB3, both had more than half of CNV losses (Figure 3C). Our data also indicated that widespread 3′ UTR shortening in LUAD might be caused by the elevated expression of polyadenylation factors through enhanced usage of PAS, consistent with findings from a study in proliferating cells (Elkon et al., 2012).

**FIGURE 4:**
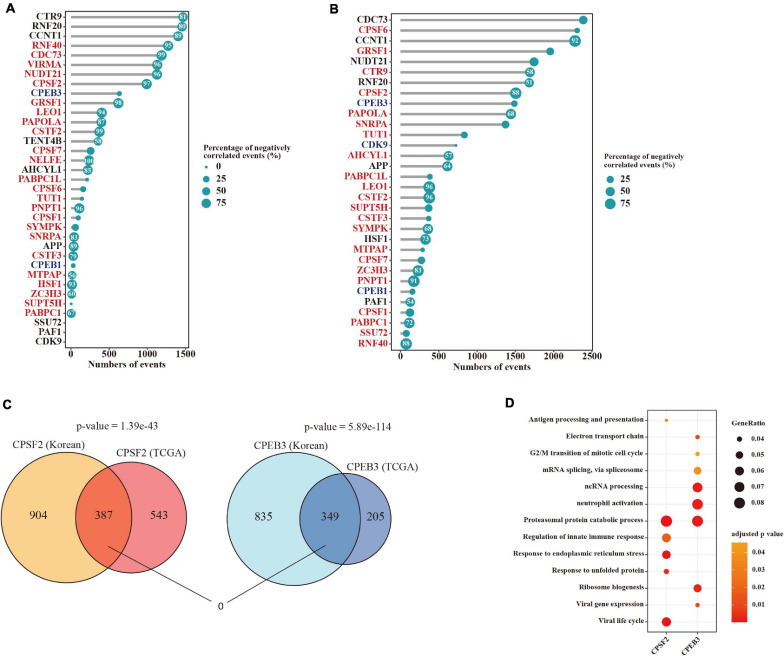
Potential mechanisms for APA regulation in LUAD. **(A)** Lollipop chart indicating the number of significantly correlated APA events with each regulator gene in the TCGA dataset. Dot size is proportional to the percentage of negatively correlated events. Numbers in the dots represent the percentages of negatively correlated events that are greater than 50%. **(B)** Lollipop chart indicating the number of significantly correlated APA events with each regulator gene in the Korea dataset. **(C)** Venn diagram showing the overlap of altered APA events correlated with CPSF2 or CPEB3 between the two datasets. **(D)** Dot plot indicating significantly enriched biological processes of events correlated with CPSF2 or CPEB3 in both two cohorts.

We further investigated the correlation between APA events and the expression levels of their regulators in tumors. Remarkably, among these APA events 44.2% (3,165/7,163) and 79.4% (5,618/7,072) of them were significantly corrected (|rho| > 0.3 and adjusted *p*-value < 0.05) with at least one factor in the TCGA and Korea datasets respectively (Figures 4A,B). Moreover, we observed strongly negative associations between 3′ UTR lengths and mRNA expression of those factors in the TCGA dataset (Figure 4A). To define certain factors dysregulated in tumors that could primarily be responsible for APA mechanism in LUAD, we filtered APA factors through those upregulated with more than half of negatively correlated events or downregulated with more than half of positively correlated events. Subsequently, CPSF2 and CPEB3 were identified that correlated with more than 500 APA events in both datasets, which could be master regulators of APA in LUAD. Moreover, 387 and 349 genes were determined to be correlated with CPSF2 and CPEB3 in both datasets respectively (Figure 4C), showing strongly significant overlaps (*p*-value = 1.39e−43 and *p*-value = 5.89e−114, hypergeometric test). Interestingly, no genes were shared by CPSF2- and CPEB3-correlated APA events (Figure 4C), suggesting that the two factors may regulate APA alternations in LUAD independently. To test it, we performed an overrepresentation analysis of biological processes for 387 and 349 genes correlated with the two factors. As shown in Figure 4D, they can both regulate the proteasomal protein catabolic process through the APA mechanism and most other processes enriched in the two factors were different.

### The Prognostic Value of APA Events and Their Regulators in LUAD

**FIGURE 5:**
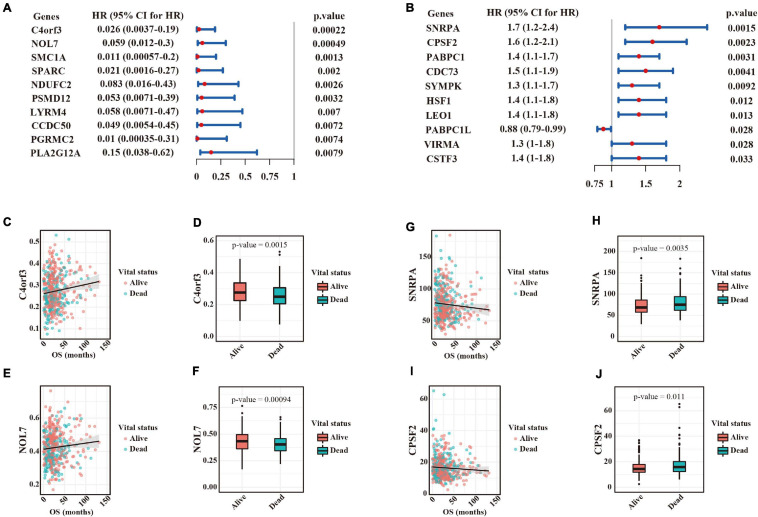
Survival-associated APA events and their regulators in LUAD. **(A)** Ranked list of the top ten survival-associated APA events according to the *p*-values calculated by the likelihood ratio test. Forest plots showing the hazard ratio and its upper and lower boundary of 95% confidence interval. Hazard ratios > 1 indicated survival risks, whereas those <1 were associated with better outcomes. **(B)** Ranked list of the top ten survival-associated APA regulators according to the *p*-values calculated by the likelihood ratio test. **(C)** Scatter plot of C4orf3 PDUI scores (*y* axis) and survival time (*x* axis). Each dot represents a tumor sample. **(D)** Comparison of C4orf3 PDUI scores between alive and dead patients. **(E)** Scatter plot of NOL7 expression (*y* axis) and survival time (*x* axis). **(F)** Comparison of NOL7 PDUI scores between alive and dead patients. **(G)** Scatter plot of SNRPA TPM values (*y* axis) and survival time (*x* axis). **(H)** Comparison of SNRPA expression between alive and dead patients. **(I)** Scatter plot of CPSF2 TPM values (*y* axis) and survival time (*x* axis). **(J)** Comparison of CPSF2 expression between alive and dead patients.

Understanding the widespread alterations in APA events and their regulators in LUAD could provide important insights for translational medicine. We performed univariate Cox regression analyses between survival time and 272 transcripts with significant 3′ UTR changes in the TCGA dataset. In total, 51 events significantly associated with survival time were identified. Notably, patients with shortened 3′ UTRs for all those events had poorer clinical outcomes, thus suggesting that use of a PAS may exacerbate LUAD malignancy. The top ten significant events are shown in Figure 5A, whose hazard ratios ranged from 0.026 to 0.15. Scatter plot and box plot (Figures 5C–F) further showed the positive association between PDUI scores and survival results (e.g., C4orf3 and NOL7). Furthermore, we focused on the associations between the expression of APA factors and survival results. Ten factors were identified to be significantly correlated with survival time (Figure 5B). Importantly, among them, a high expression of nine genes that were upregulated in tumors was associated with poor prognosis of patients with LUAD. Scatter plot and box plot (Figures 5G–J) further showed the negative association between the expression levels of most APA factors and survival results (e.g., SNRPA and CPSF2).

### Examination of APA Factors Mediating Heterogeneity of Proximal PAS Usage Identifies LUAD Subtypes With Distinct Clinical and Molecular Features

**FIGURE 6:**
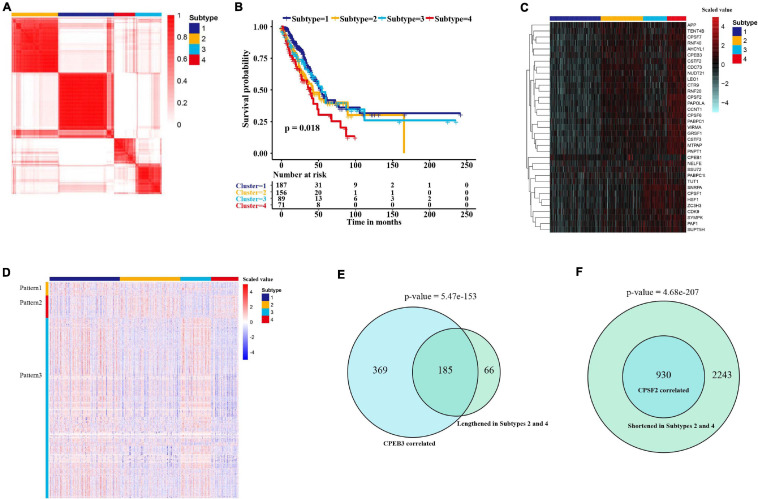
Expression heterogeneity of APA factors reveals LUAD subtypes with distinct APA patterns and clinical features. **(A)** Consensus clustering of patients (*n* = 512) based on expression of APA factors identifies four subtypes in LUAD. The color from white to red represents the consistency ranging from 0 to 1. **(B)** Kaplan–Meier survival plot of patients grouped by global expression patterns of APA regulators. The survival difference was determined by the log-rank test. **(C)** Heat map of 35 APA factors showing the difference among the four subtypes. The color indicates the scaled expression value (red, high; blue, low). **(D)** Heat map of 3,730 APA events displaying the differences among the four subtypes. The color indicates the scaled PDUI value (red, high; blue, low). **(E)** Venn diagram showing the overlap between altered APA events correlated with CPSF2 and events lengthened in subgroups 2 and 4. **(F)** Venn diagram showing the overlap between altered APA events correlated with CPEB3 and events shortened in subgroups 2 and 4.

Next, we explored whether the expression of APA factors might contribute to the stratification of LUAD. According to the expression pattern of APA regulators, we identified four subtypes of 512 patients in the TCGA cohort through consensus clustering (Figure 6A). The optimal number of subtypes was determined by an empirical CDF plot (Supplementary Figures 3A,B). The four subtypes displayed significant differences in overall survival (Figure 6B). Among them, subtype 4 consisted of 50 patients with the highest expression of APA factors, who had the worst survival results (Figures 6B,C). To investigate APA factors mediating heterogeneity of proximal PAS usage in tumors, we further compared the 3′ UTR differences among the four subtypes. In agreement with the expression levels of APA regulators, subtype 1 showed the greatest usage of distal 3′ UTRs whereas subtype 4 displayed the greatest proximal APAs (Supplementary Figure 3C). Moreover, we compared the 3′ UTR lengths and miRNA-binding sites between the events significantly shortened in subtype 4 (adjusted *p*-value < 0.05) and those below the threshold. As with the differentially regulated APA events, 3′ UTR-shortened transcripts in subtype 4 showed longer 3′ UTR lengths and greater miRNA-binding sites (Supplementary Figures 3D,E), suggesting that 3′ UTR shortening-mediated loss of miRNA-binding sites was associated with LUAD aggressiveness. To explore whether any distinct APA patterns can be seen among the four subtypes of LUAD, we further investigated 3,731 significantly different APA events (Kruskal–Wallis test, adjusted *p*-value < 0.001). As shown in Figure 6D, three distinct patterns that may be regulated by specific factors were observed in four groups. The APA events with pattern 1 showed shorter 3′ UTR subtypes 3 and 4, which may be regulated by the factors upregulated in these two subtypes. Intriguingly, we found that pattern 2 showed longer 3′ UTR in subtypes 2 and 4. We hypothesized that pattern 2 could be caused by CPEB3 that was upregulated in those two subtypes. To test it, we compared this pattern with CPEB3 positively correlated events. As shown in Figure 6E, among 251 lengthened genes, 73.7% (185) may depend on the expression of CPEB3. Pattern 3 consisted of the largest numbers of APA events that were shortened in subtypes 2 and 4, which may be caused by the factors upregulated in these two subtypes. In addition, APA events negatively correlated with CPSF2 which we identified as a possible master regulator were all in pattern 3 (Figure 6F).

**FIGURE 7:**
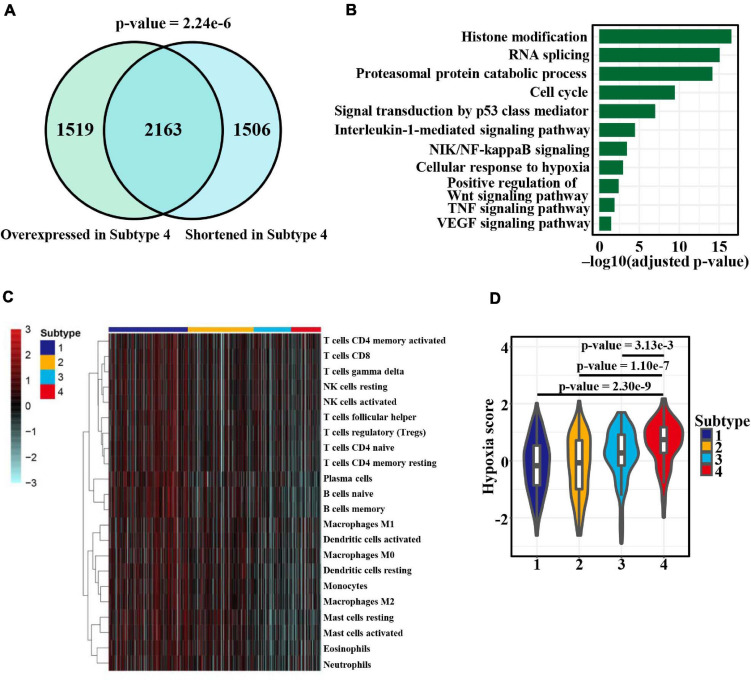
APA heterogeneity contributing to gene expression and TME differences among patients with LUAD. **(A)** Venn diagram comparison between genes with shortened 3′ UTRs and those upregulated in subtype 4 (adjusted *p*-value < 0.05, one-sided Wilcoxon rank-sum test). The color from white to red represents the consistency ranging from 0 to 1. **(B)** Significantly enriched (adjusted *p*-value < 0.05, hypergeometric test) GO terms and KEGG pathways in the 2,163 overlapping genes. **(C)** Heat map of 22 immune cell subsets calculated with ssGSEA, indicating the TME differences among the four subtypes. The color indicates the normalized score value (red, high; blue, low). **(D)** Differences in hypoxia scores among the four groups. Statistical differences were determined by the Wilcoxon rank-sum test.

To investigate the impact of APA heterogeneity on gene expression, we focused on subtype 4 exhibiting the greatest APA changes. We found that among 3,669 shortened genes, 2,163 (*p*-value = 2.24e−6, hypergeometric test) showed a significantly higher expression level in subtype 4 (Figure 7A). We further explored the functional implications of the gene expression heterogeneity among the four subtypes mediated by APA events. GO and KEGG pathway enrichment analysis of 2,163 overlapping genes identified several highly enriched GO terms: histone modification, RNA splicing, proteasomal process, and cell cycle (Figure 7B). Similar biological processes have been observed in pan-cancer correlation analysis (Xiang et al., 2018), and our results further suggested APA regulation of those functions. Remarkably, we also found enriched pathways related to immune and hypoxia such as NIK/NF-κB, Wnt, and TNF signaling (Figure 7B). Thus, the infiltration levels of 22 immune cells and hypoxia status in patients were estimated by using ssGSEA based on the previous reported gene signatures (Newman et al., 2015; Ye et al., 2019). Strikingly, the four subtypes displayed marked differences in immune and hypoxia status (Figures 7C,D). Subtype 1, with the greatest distal PAS usage, showed the highest innate and adaptive immune cell infiltration and the lowest hypoxia score (Figures 7C,D). Overall, these results indicated the role of APA in shaping the tumor microenvironment (TME) or vice versa.

### Heterogeneity of Proximal PAS Usage of Metabolic Genes in LUAD Patients

**FIGURE 8:**
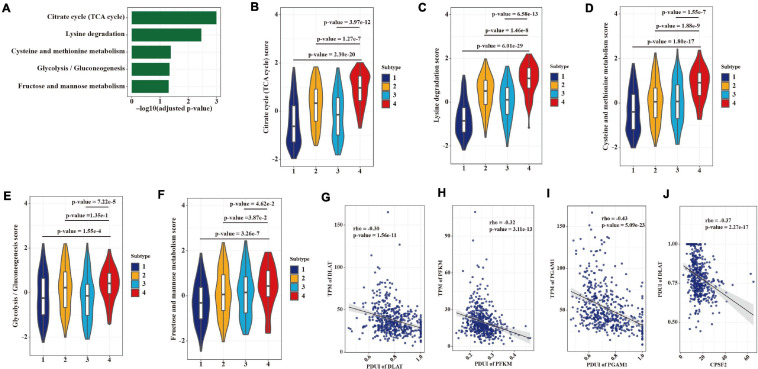
APA heterogeneity contributing to metabolic gene expression differences in LUAD. **(A)** Significantly enriched (adjusted *p*-value < 0.05, hypergeometric test) metabolic pathways in the 2,163 overlapping genes. **(B–F)** Differences in scores of metabolic pathways among the four groups. Statistical differences were determined by the Wilcoxon rank-sum test. **(G)** Correlation between the expression level and the APA event of DLAT. **(H)** Correlation between the expression level and the APA event of PFKM. **(I)** Correlation between the expression level and the APA event of PGAM1. **(J)** Correlation between the expression level of CPSF2 and the APA event of DLAT.

We also found that gene expression heterogeneity among LUAD patients mediated by APA events was enriched in five metabolic pathways including citrate cycle (TCA cycle), lysine degradation, cysteine and methionine metabolism, glycolysis (gluconeogenesis), and fructose and mannose metabolism (Figure 8A). Therefore, we compared the NESs of five pathways among four subtypes. As expected, subtype 4, with the greatest usage of proximal PAS, showed the highest score in all five metabolic pathways (Figures 8B–F). To examine whether this heterogeneity is regulated by APA mechanism, we conducted the correlations between expression levels of genes in the glycolytic pathway and their 3′ UTR lengths. DLAT, PFKM, and PGAM1 were reported can promote cancer cell growth through the glycolytic pathway (Tang et al., 2012; Goh et al., 2015; Huang et al., 2019). As shown in Figures 8G–I, DLAT, PFKM, and PGAM1 were all negatively correlated with their PDUI values. Moreover, we found that CPSF2 may regulate APA events of metabolic genes like DLAT (Figure 8J).

## Discussion

Based on the large-scale RNA-seq data from two cohorts, we provided a systemic and specific portrait of the APA landscape in LUAD. In agreement with previous studies (Xia et al., 2014; Xiang et al., 2018), our analyses revealed global shortening of APA in tumor samples when compared with paired controls. Notably, we found high consistency in APA alterations between the two datasets, a result previously unnoticed in pan-cancer or single-tumor-type analyses. Moreover, genes with significantly changed 3′ UTRs were enriched in locations of cell membrane and some organelle membranes, including those of lysosomes, vacuoles, and late endosomes. A novel mechanism in which alternative 3′ UTR isoforms of membrane genes can determine their subcellular protein localization and function has been identified in a previous study (Berkovits and Mayr, 2015). CD47, a well-established cell surface molecule, can produce alternative 3′ UTR isoforms that localize to different cellular compartments and show opposite functions in cell survival and cell migration (Berkovits and Mayr, 2015). Our analyses suggest that alternations of 3′ UTR lengths in membrane-associated genes may promote cancer cell growth through APA-dependent protein localization. 3′ UTR shortening-mediated miRNA binding loss has been found to affect the expression levels of these genes (Venkat et al., 2020). We observed a considerable number of genes upregulated in LUAD after shortening of their 3′ UTRs, but this result was not statistically significant when compared with the global pattern of DEGs, which indicates that APA is only one of the multiple mechanisms that govern mRNA expression levels (Venkat et al., 2020).

The regulation of alternative 3′ UTR usage in LUAD remains unclear. Our analyses indicate that most APA factors are overexpressed and negatively correlated with distal PAS usage in LUAD. CSTF2 has been recognized as the key factor that induces 3′ UTR shortening in pan-cancer analysis (Xia et al., 2014) and has been implicated in contributing to carcinogenesis of the bladder (Chen et al., 2018), breast (Akman et al., 2015), and lung (Aragaki et al., 2011). In contrast, we found that several APA factors may act as master regulators in LUAD, such as RNF40, CDC73, and VIRMA, which are not core proteins in the polyadenylation machinery. The methyltransferase component VIRMA facilitates the selection of proximal PAS through preferential m6A mRNA methylation in the 3′ UTR and near the stop codon (Yue et al., 2018). Indeed, depletion of VIRMA or METTL3 elicits global lengthening of APA events in the HeLa cell line (Yue et al., 2018). Our analysis further indicated that a high expression level of VIRMA is associated with poor survival outcomes in patients with LUAD. These findings provide a possibility that VIRMA may serve as an oncogene in LUAD that negatively regulates the 3′ UTR lengths of cancer-associated genes through m6A mRNA methylation to enhance tumorigenicity. In addition to the factors that induce 3′ UTR shortening, a previous study has revealed PABPN1 as a master regulator that promotes distal PAS usage in pan-cancer analyses including LUAD (Xiang et al., 2018). We also found that PABPN1 positively correlates with 17.6% of APA events in the Korean LUAD cohort (data not shown). Our analysis identified two genes, CPEB1 and CPEB3, which were both downregulated in the two datasets. Compared with most upregulated genes, CPEB3 is more positively correlated with APA events in tumors, thus suggesting that its regulation of preferential distal poly(A) site usage may be inhibited in LUAD. We directly calculated correlations between APA events and factors to define the potential regulations of those factors in LUAD. This analysis has a limitation in that identified regulators may be dependent on other co-expressed factors. Therefore, further experimental validation is necessary to explore the molecular mechanisms of APA regulations in LUAD. Together, our results suggest that dysregulated APA factors in LUAD may be considered as potential biomarkers and therapeutic targets, which should be further confirmed through additional experiments.

Several studies have shown the prognostic power of APA events in different cancers (Xia et al., 2014; Venkat et al., 2020). Our analyses further revealed that the patients with shorter 3′ UTR lengths show poor survival in LUAD. Some APA events from our analyses provided noteworthy biological and clinical insights. SMC1A, a core cohesin gene, has been reported to promote tumor development in some types of human cancers (Pan et al., 2016; Zhou et al., 2017; Sarogni et al., 2019). Our results showed that the 3′ UTR of SMC1A is shortened in tumors and is significantly associated with clinical prognosis, thus providing a potential mechanism through which overexpression of SMC1A in human cancers may be contributed by marked shortening of its 3′ UTR. Expression levels of SPARC in patients with NSCLC are associated with disease diagnosis and prognosis (Koukourakis et al., 2003; Huang et al., 2012; Andriani et al., 2018). Our analyses further indicate that different poly(A) site usage of SPARC may also serve as a diagnostic and prognostic factor.

By stratification of patients with LUAD, we identified that heterogeneity in PAS usage among tumors can be explained by the mRNA expression levels of APA factors. Furthermore, 3′ UTR differences among the four subtypes considerably affected the specific mRNA transcriptome. Previous studies have shown that regulators of 3′ end processing can influence the 3′ UTR of genes in the Wnt/β-catenin and NF-κB signaling pathways, thereby determining the cancer phenotype (Ogorodnikov et al., 2018; Xiong et al., 2019). Consistent with these findings, our data underscore the crucial roles of APA factors in governing the patient-specific APA alternations, a process tightly associated with the activation of oncogenic pathways. Besides demonstrating the influence on the transcriptome in patients, our analyses suggest that 3′ UTR changes strikingly affect tumor immune and hypoxia status or vice versa. Patients with longer 3′ UTRs in global APA characterization showed higher immune and lower hypoxia scores. This finding may provide insights into strategies for potential cancer therapies targeting tumor immunity or hypoxia. Proliferating cells expressing mRNAs with shortened 3′ UTR has long been recognized (Sandberg et al., 2008). Our results suggest that APA may contribute to the altered levels of metabolic genes which in turn create a TME that promote their survival and propagation.

In summary, we presented the comprehensive landscape of 3′ UTR in LUAD and highlighted 113 recurrent APA alterations and specific factors especially two key regulators, CPSF2 and CPEB3, regulating APA patterns. Consistent with previous analyses in other cancer types, 3′ UTR shortening is frequently associated with tumor occurrence in APA events, and it may contribute to elevated gene expression through loss of miRNA-binding sites. Moreover, APA events and their regulators were found to be useful for prognosis and cancer stratification in LUAD. The resources provided herein should be valuable for understanding and exploring alternative 3′ UTR isoforms in LUAD and are expected to promote precision medicine in the future.

## Data Availability Statement

The original contributions presented in the study are included in the article/Supplementary Material, further inquiries can be directed to the corresponding author/s.

## Author Contributions

FW, GZ, and QS proposed and designed the project. YZ, LS, and QS performed the data collection and analyses. YZ and FW wrote the manuscript. FW and GZ revised the manuscript. All authors read and approved the final manuscript.

## Conflict of Interest

The authors declare that the research was conducted in the absence of any commercial or financial relationships that could be construed as a potential conflict of interest.

## Footnotes

1 https://portal.gdc.cancer.gov/

2 https://github.com/ZhengXia/DaPars

3 http://www.cbioportal.org/

4 https://cibersort.stanford.edu/

## References

[B1] Agarwal V. Bell G. W. Nam J. W. Bartel D. P. (2015). Predicting effective microRNA target sites in mammalian mRNAs. *Elife* 4:e05005. 10.7554/eLife.05005 PMC4532895 26267216

[B2] Akman H. B. Oyken M. Tuncer T. Can T. Erson-Bensan A. E. (2015). 3′UTR shortening and EGF signaling: implications for breast cancer. *Hum. Mol. Genet.* 24 6910–6920. 26395459 10.1093/hmg/ddv391

[B3] Allen M. Bird C. Feng W. Liu G. Li W. Perrone-Bizzozero N. I. (2013). HuD promotes BDNF expression in brain neurons via selective stabilization of the BDNF long 3′UTR mRNA. *PLoS One* 8:e55718. 10.1371/journal.pone.0055718 23383270 PMC3561324

[B4] Andriani F. Landoni E. Mensah M. Facchinetti F. Miceli R. Tagliabue E. (2018). Diagnostic role of circulating extracellular matrix-related proteins in non-small cell lung cancer. *BMC Cancer* 18:899. 10.1186/s12885-018-4772-0 PMC6145327 30227835

[B5] Aragaki M. Takahashi K. Akiyama H. Tsuchiya E. Kondo S. Nakamura Y. (2011). Characterization of a cleavage stimulation factor, 3′ pre-RNA, subunit 2, 64 kDa (CSTF2) as a therapeutic target for lung cancer. *Clin. Cancer Res.* 17 5889–5900. 10.1158/1078-0432.ccr-11-0240 21813631

[B6] Arake D. T. L. Pulos-Holmes M. C. Floor S. N. Cate J. (2019). PTBP1 mRNA isoforms and regulation of their translation. *RNA* 25 1324–1336. 10.1261/rna.070193.118 31263002 PMC6800477

[B7] Arefeen A. Liu J. Xiao X. Jiang T. (2018). TAPAS: tool for alternative polyadenylation site analysis. *Bioinformatics* 34 2521–2529. 10.1093/bioinformatics/bty110 30052912 PMC6454472

[B8] Berkovits B. D. Mayr C. (2015). Alternative 3′ UTRs act as scaffolds to regulate membrane protein localization. *Nature* 522 363–367. 10.1038/nature14321 25896326 PMC4697748

[B9] Chen X. Zhang J. X. Luo J. H. Wu S. Yuan G. J. Ma N. F. (2018). CSTF2-induced shortening of the RAC1 3′UTR promotes the pathogenesis of Urothelial Carcinoma of the Bladder. *Cancer Res.* 78 5848–5862. 30143523 10.1158/0008-5472.CAN-18-0822

[B10] Chu Y. Elrod N. Wang C. Li L. Chen T. Routh A. (2019). Nudt21 regulates the alternative polyadenylation of Pak1 and is predictive in the prognosis of glioblastoma patients. *Oncogene* 38 4154–4168. 10.1038/s41388-019-0714-9 30705404 PMC6533131

[B11] Derti A. Garrett-Engele P. Macisaac K. D. Stevens R. C. Sriram S. Chen R. (2012). A quantitative atlas of polyadenylation in five mammals. *Genome Res.* 22 1173–1183. 10.1101/gr.132563.111 22454233 PMC3371698

[B12] Elkon R. Drost J. van Haaften G. Jenal M. Schrier M. Oude V. J. (2012). E2F mediates enhanced alternative polyadenylation in proliferation. *Genome Biol.* 13:R59. 10.1186/gb-2012-13-7-r59 PMC3491381 22747694

[B13] Esfahani M. S. Lee L. J. Jeon Y. J. Flynn R. A. Stehr H. Hui A. B. (2019). Functional significance of U2AF1 S34F mutations in lung adenocarcinomas. *Nat. Commun.* 10:5712. 10.1038/s41467-019-13392-y PMC6911043 31836708

[B14] Fischl H. Neve J. Wang Z. Patel R. Louey A. Tian B. (2019). hnRNPC regulates cancer-specific alternative cleavage and polyadenylation profiles. *Nucleic Acids Res.* 47 7580–7591. 10.1093/nar/gkz461 31147722 PMC6698646

[B15] Gao J. Aksoy B. A. Dogrusoz U. Dresdner G. Gross B. Sumer S. O. (2013). Integrative analysis of complex cancer genomics and clinical profiles using the cBioPortal. *Sci. Signal.* 6:l1. 10.1126/scisignal.2004088 PMC4160307 23550210

[B16] Goh W. Q. Ow G. S. Kuznetsov V. A. Chong S. Lim Y. P. (2015). DLAT subunit of the pyruvate dehydrogenase complex is upregulated in gastric cancer-implications in cancer therapy. *Am. J. Transl. Res.* 7 1140–1151. 26279757 PMC4532746

[B17] Gruber A. J. Zavolan M. (2019). Alternative cleavage and polyadenylation in health and disease. *Nat. Rev. Genet.* 20 599–614. 10.1038/s41576-019-0145-z 31267064

[B18] Halees A. S. El-Badrawi R. Khabar K. S. (2008). ARED Organism: expansion of ARED reveals AU-rich element cluster variations between human and mouse. *Nucleic Acids Res.* 36 D137–D140. 17984078 10.1093/nar/gkm959 PMC2238997

[B19] Hanzelmann S. Castelo R. Guinney J. (2013). GSVA: gene set variation analysis for microarray and RNA-seq data. *BMC Bioinformatics* 14:7. 10.1186/1471-2105-14-7 23323831 PMC3618321

[B20] Herbst R. S. Morgensztern D. Boshoff C. (2018). The biology and management of non-small cell lung cancer. *Nature* 553 446–454. 29364287 10.1038/nature25183

[B21] Huang K. Liang Q. Zhou Y. Jiang L. L. Gu W. M. Luo M. Y. (2019). A Novel allosteric inhibitor of phosphoglycerate mutase 1 suppresses growth and metastasis of non-small-cell lung cancer. *Cell Metab.* 30 1107–1119. 10.1016/j.cmet.2019.09.014 31607564

[B22] Huang Y. Zhang J. Zhao Y. Y. Jiang W. Xue C. Xu F. (2012). SPARC expression and prognostic value in non-small cell lung cancer. *Chin. J. Cancer* 31 541–548. 23114088 10.5732/cjc.012.10212 PMC3777514

[B23] Koukourakis M. I. Giatromanolaki A. Brekken R. A. Sivridis E. Gatter K. C. Harris A. L. (2003). Enhanced expression of SPARC/osteonectin in the tumor-associated stroma of non-small cell lung cancer is correlated with markers of hypoxia/acidity and with poor prognosis of patients. *Cancer Res.* 63 5376–5380. 14500371

[B24] Li Y. Xiao J. Bai J. Tian Y. Qu Y. Chen X. (2019). Molecular characterization and clinical relevance of m(6)A regulators across 33 cancer types. *Mol. Cancer* 18:137. 10.1186/s12943-019-1066-3 PMC6744659 31521193

[B25] Lin Y. Li Z. Ozsolak F. Kim S. W. Arango-Argoty G. Liu T. T. (2012). An in-depth map of polyadenylation sites in cancer. *Nucleic Acids Res.* 40 8460–8471. 10.1093/nar/gks637 22753024 PMC3458571

[B26] Liu Y. Sun J. Zhao M. (2017). ONGene: a literature-based database for human oncogenes. *J. Genet. Genomics* 44 119–121. 10.1016/j.jgg.2016.12.004 28162959

[B27] Masamha C. P. Xia Z. Yang J. Albrecht T. R. Li M. Shyu A. B. (2014). CFIm25 links alternative polyadenylation to glioblastoma tumour suppression. *Nature* 510 412–416. 10.1038/nature13261 24814343 PMC4128630

[B28] Mayr C. Bartel D. P. (2009). Widespread shortening of 3′UTRs by alternative cleavage and polyadenylation activates oncogenes in cancer cells. *Cell* 138 673–684. 10.1016/j.cell.2009.06.016 19703394 PMC2819821

[B29] Newman A. M. Liu C. L. Green M. R. Gentles A. J. Feng W. Xu Y. (2015). Robust enumeration of cell subsets from tissue expression profiles. *Nat. Methods* 12 453–457. 10.1038/nmeth.3337 25822800 PMC4739640

[B30] Ogorodnikov A. Levin M. Tattikota S. Tokalov S. Hoque M. Scherzinger D. (2018). Transcriptome 3′end organization by PCF11 links alternative polyadenylation to formation and neuronal differentiation of neuroblastoma. *Nat. Commun.* 9:5331. 10.1038/s41467-018-07580-5 PMC6294251 30552333

[B31] Pan X. W. Gan S. S. Ye J. Q. Fan Y. H. Hong U. Chu C. M. (2016). SMC1A promotes growth and migration of prostate cancer in vitro and in vivo. *Int. J. Oncol.* 49 1963–1972. 10.3892/ijo.2016.3697 27667360

[B32] Park H. J. Ji P. Kim S. Xia Z. Rodriguez B. Li L. (2018). 3′ UTR shortening represses tumor-suppressor genes in trans by disrupting ceRNA crosstalk. *Nat. Genet.* 50 783–789. 10.1038/s41588-018-0118-8 29785014 PMC6689271

[B33] Robinson M. D. McCarthy D. J. Smyth G. K. (2010). edgeR: a bioconductor package for differential expression analysis of digital gene expression data. *Bioinformatics* 26 139–140. 10.1093/bioinformatics/btp616 19910308 PMC2796818

[B34] Sandberg R. Neilson J. R. Sarma A. Sharp P. A. Burge C. B. (2008). Proliferating cells express mRNAs with shortened 3′ untranslated regions and fewer microRNA target sites. *Science* 320 1643–1647. 10.1126/science.1155390 18566288 PMC2587246

[B35] Sarogni P. Palumbo O. Servadio A. Astigiano S. D’Alessio B. Gatti V. (2019). Overexpression of the cohesin-core subunit SMC1A contributes to colorectal cancer development. *J. Exp. Clin. Cancer Res.* 38:108. 10.1186/s13046-019-1116-0 PMC6397456 30823889

[B36] Sebestyen E. Singh B. Minana B. Pages A. Mateo F. Pujana M. A. (2016). Large-scale analysis of genome and transcriptome alterations in multiple tumors unveils novel cancer-relevant splicing networks. *Genome Res.* 26 732–744. 10.1101/gr.199935.115 27197215 PMC4889968

[B37] Tan S. Li H. Zhang W. Shao Y. Liu Y. Guan H. (2018). NUDT21 negatively regulates PSMB2 and CXXC5 by alternative polyadenylation and contributes to hepatocellular carcinoma suppression. *Oncogene* 37 4887–4900. 10.1038/s41388-018-0280-6 29780166

[B38] Tang H. Lee M. Sharpe O. Salamone L. Noonan E. J. Hoang C. D. (2012). Oxidative stress-responsive microRNA-320 regulates glycolysis in diverse biological systems. *FASEB J.* 26 4710–4721. 10.1096/fj.11-197467 22767230 PMC3475252

[B39] Tian B. Manley J. L. (2017). Alternative polyadenylation of mRNA precursors. *Nat. Rev. Mol. Cell Biol.* 18 18–30. 10.1038/nrm.2016.116 27677860 PMC5483950

[B40] Venkat S. Tisdale A. A. Schwarz J. R. Alahmari A. A. Maurer H. C. Olive K. P. (2020). Alternative polyadenylation drives oncogenic gene expression in pancreatic ductal adenocarcinoma. *Genome Res.* 30 347–360. 10.1101/gr.257550.119 32029502 PMC7111527

[B41] Wilkerson M. D. Hayes D. N. (2010). Consensusclusterplus: a class discovery tool with confidence assessments and item tracking. *Bioinformatics* 26 1572–1573. 10.1093/bioinformatics/btq170 20427518 PMC2881355

[B42] Xia Z. Donehower L. A. Cooper T. A. Neilson J. R. Wheeler D. A. Wagner E. J. (2014). Dynamic analyses of alternative polyadenylation from RNA-seq reveal a 3′-UTR landscape across seven tumour types. *Nat. Commun.* 5:5274. 10.1038/ncomms6274 PMC4467577 25409906

[B43] Xiang Y. Ye Y. Lou Y. Yang Y. Cai C. Zhang Z. (2018). Comprehensive characterization of alternative polyadenylation in human cancer. *J. Natl. Cancer Inst.* 110 379–389. 10.1093/jnci/djx223 29106591 PMC6059203

[B44] Xiong M. Chen L. Zhou L. Ding Y. Kazobinka G. Chen Z. (2019). NUDT21 inhibits bladder cancer progression through ANXA2 and LIMK2 by alternative polyadenylation. *Theranostics* 9 7156–7167. 10.7150/thno.36030 31695759 PMC6831288

[B45] Ye C. Long Y. Ji G. Li Q. Q. Wu X. (2018). APAtrap: identification and quantification of alternative polyadenylation sites from RNA-seq data. *Bioinformatics* 34 1841–1849. 10.1093/bioinformatics/bty029 29360928

[B46] Ye Y. Hu Q. Chen H. Liang K. Yuan Y. Xiang Y. (2019). Characterization of hypoxia-associated molecular features to aid hypoxia-targeted therapy. *Nat. Metab.* 1 431–444. 10.1038/s42255-019-0045-8 31984309 PMC6980239

[B47] Yu G. Wang L. G. Han Y. He Q. Y. (2012). clusterProfiler: an R package for comparing biological themes among gene clusters. *OMICS* 16 284–287. 10.1089/omi.2011.0118 22455463 PMC3339379

[B48] Yue Y. Liu J. Cui X. Cao J. Luo G. Zhang Z. (2018). VIRMA mediates preferential m(6)A mRNA methylation in 3′UTR and near stop codon and associates with alternative polyadenylation. *Cell Discov.* 4:10. 10.1038/s41421-018-0019-0 PMC5826926 29507755

[B49] Zappa C. Mousa S. A. (2016). Non-small cell lung cancer: current treatment and future advances. *Transl. Lung Cancer Res.* 5 288–300. 10.21037/tlcr.2016.06.07 27413711 PMC4931124

[B50] Zhao M. Kim P. Mitra R. Zhao J. Zhao Z. (2016). TSGene 2.0: an updated literature-based knowledgebase for tumor suppressor genes. *Nucleic Acids Res.* 44 D1023–D1031. 26590405 10.1093/nar/gkv1268 PMC4702895

[B51] Zhou P. Xiao N. Wang J. Wang Z. Zheng S. Shan S. (2017). SMC1A recruits tumor-associated-fibroblasts (TAFs) and promotes colorectal cancer metastasis. *Cancer Lett.* 385 39–45. 10.1016/j.canlet.2016.10.041 27826041

