# Supplementary material for: Comprehensive Analysis of APA Events and Their Association With Tumor Microenvironment in Lung Adenocarcinoma
Source: Front Genet. 2021 Mar 18;12:645360. doi: 10.3389/fgene.2021.645360 (PMC8012674; doi:10.3389/fgene.2021.645360)
Supplement: Supplementary file 1 [file Data_Sheet_1.doc]

**Comprehensive Analysis of APA Events and Their Association With Tumor Microenvironment in Lung Adenocarcinoma**

Yuchu Zhang^1^, Libing Shen^2^, Qili Shi^3*^, Guofang Zhao^2*^, Fajiu Wang^2*^

Authors Address Information

^1^Department of Intensive Care Medicine, HwaMei Hospital, University of Chinese Academy of Sciences, Ningbo 315010, P.R. China

^2^Department of Cardiothoracic Surgery, HwaMei Hospital, University of Chinese Academy of Sciences, Ningbo 315010, P.R. China

^3^Fudan University Shanghai Cancer Center and Institutes of Biomedical Sciences,

Shanghai Medical College, Fudan University, Shanghai 200032, P.R. China

* Corresponding authors.

1. mails: wfjwyt@163.com, [qili.shi@hotmail.com](mailto:linjie1992@zju.edu.cn).

**
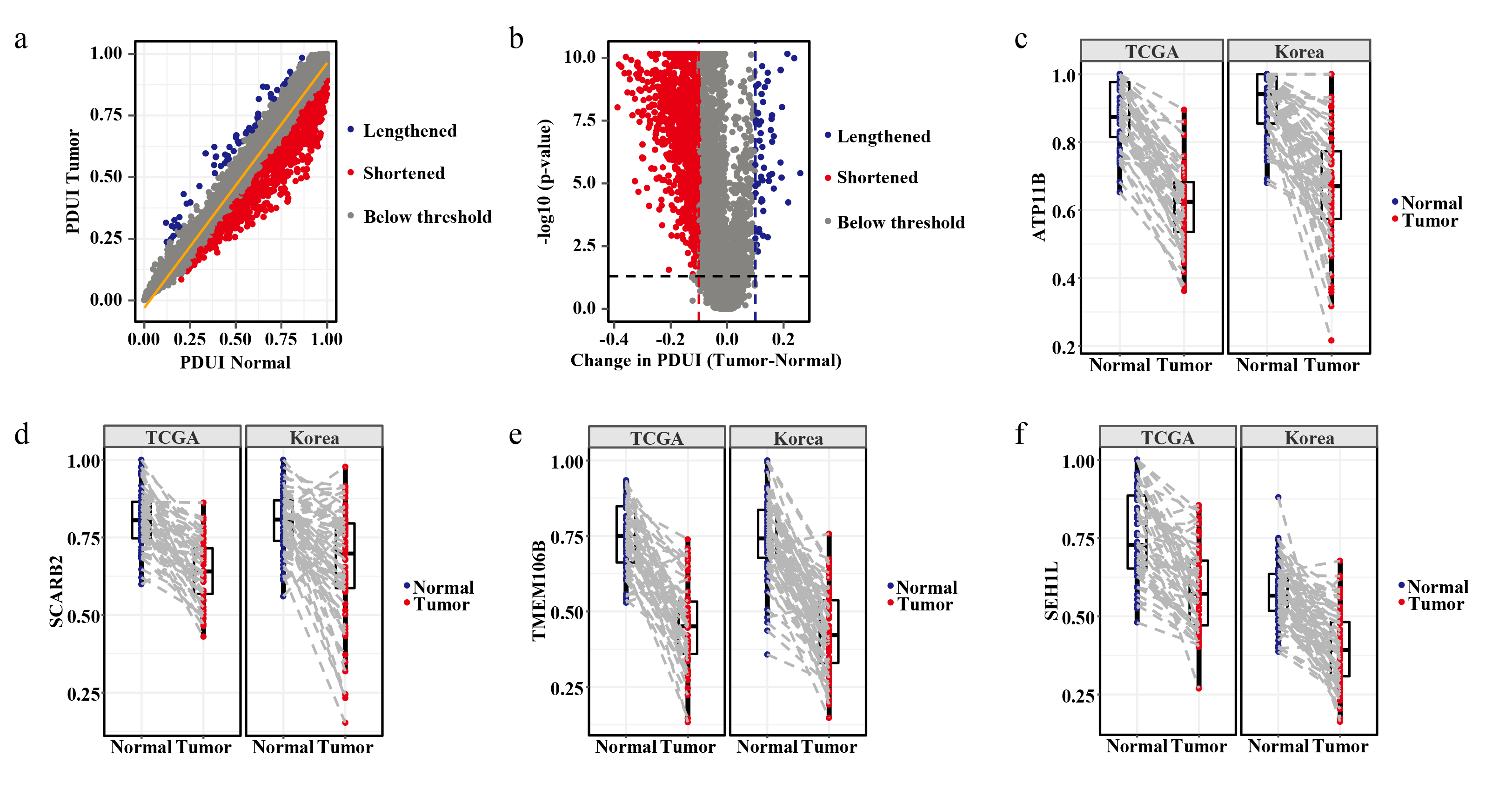
**

**Supplementary Fig. 1** Comprehensive characterization of APA changes in LUAD.

**a** Scatterplot of PDUIs in tumor and normal samples from the Korean cohort. Transcripts that were significantly (FDR < 0.05 and |ΔPDUI| > 0.1) shortened and lengthened are indicated in red and blue, respectively, whereas those below threshold are gray. **b** Volcano plot showing the significantly altered APA events in the Korean cohort. **c-f** Boxpots of significantly changed transcripts in tumors located in membranes.

**
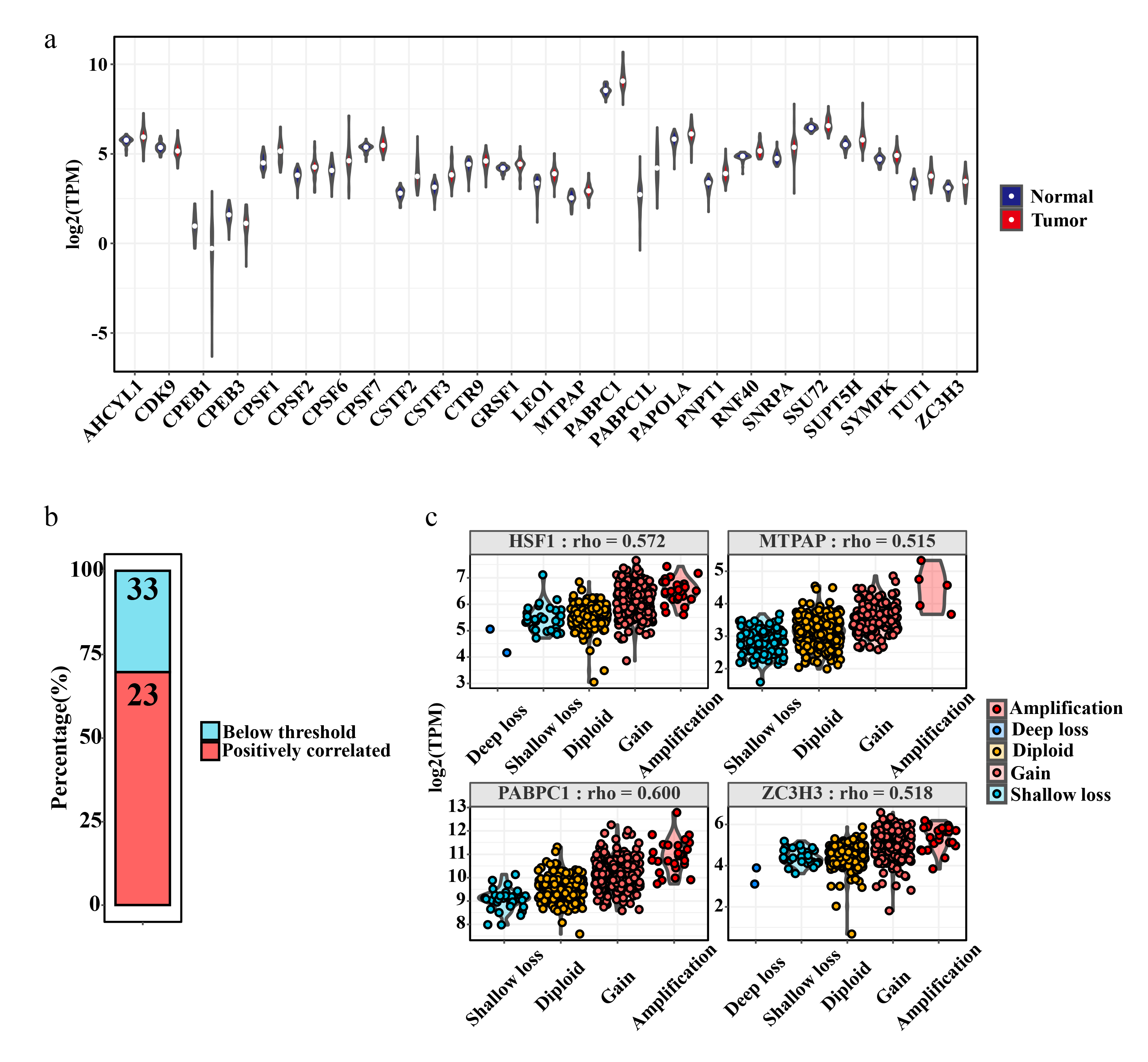
**

**Supplementary Fig. 2** CNV and expression alterations of APA regulators.

**a** Violin plot showing the expression of 25 significantly dysregulated APA factors between tumor (red) and adjacent normal (blue) samples in the Korean cohort. **b** Of the 33 APA regulators in the TCGA cohort with CNV changes, 23 are positively correlated with mRNA expression. **c** Four examples of positively correlated APA regulators between CNV and mRNA expression.


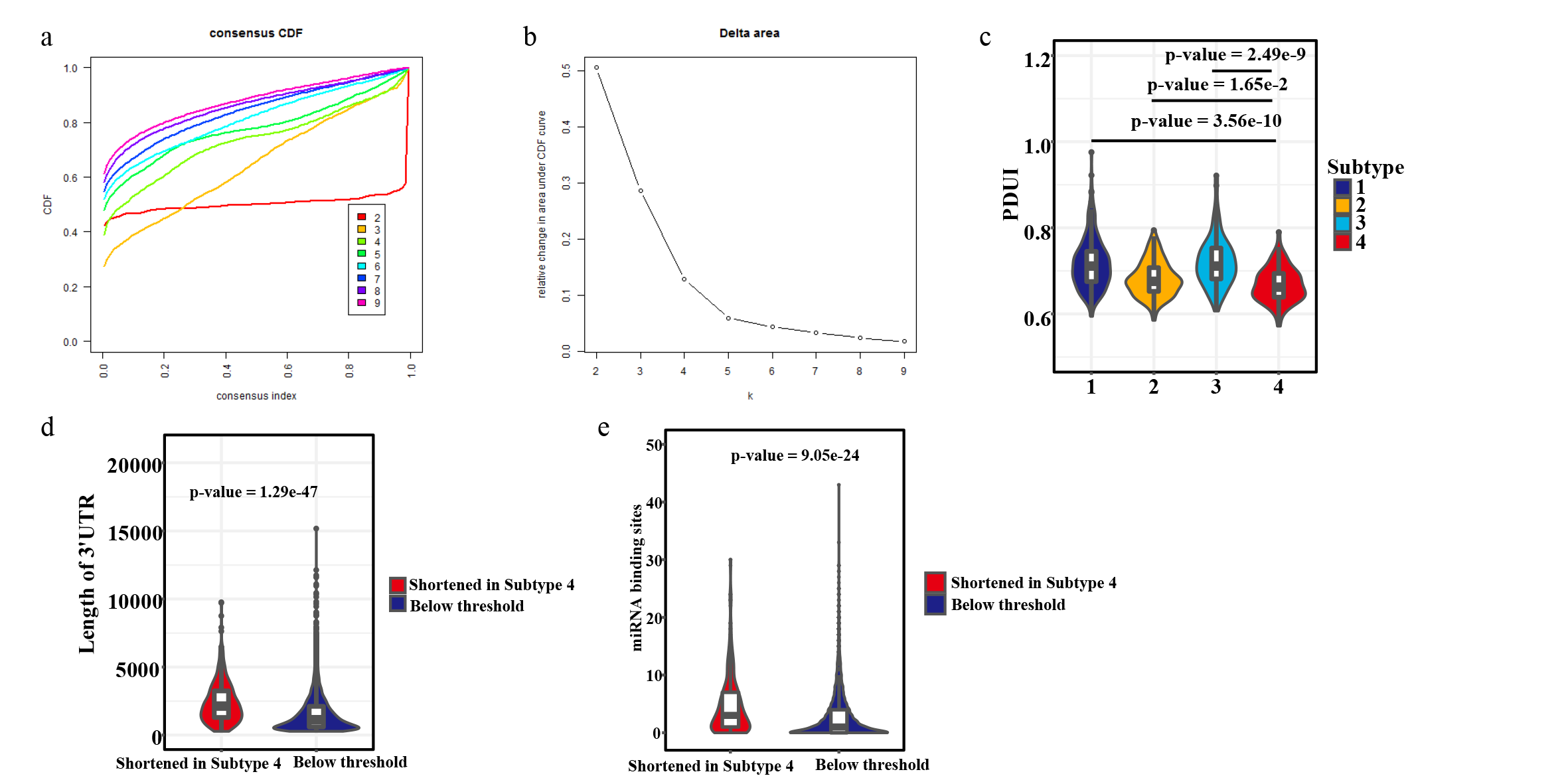


**Supplementary Fig. 3** Consensus clustering of patients with LUAD (n = 512), on the basis of the expression values of APA factors. **a** CDF plots of consensus clusters in the range k = 2, 3, . . . , 9. **b** Delta area plot showing the relative change in the area under the CDF curve when cluster k increases. **c** Comparison of PDUI values among the four groups. Statistical differences were determined by the Wilcoxon rank-sum test. **d** Significantly shortened (FDR < 0.05) transcripts in cluster 4 have longer 3´UTR lengths than do other transcripts detected in tumors that did not pass the threshold. The p-values were calculated with a Wilcoxon rank-sum test. **e** Violin plot indicating that significantly shortened transcripts in cluster 4 have more miRNA binding sites in the 3´UTR region than that of others below the threshold. Statistical differences were determined by the Wilcoxon rank-sum test.
